# *Abcd2* Is a Strong Modifier of the Metabolic Impairments in Peritoneal Macrophages of *Abcd1*-Deficient Mice

**DOI:** 10.1371/journal.pone.0108655

**Published:** 2014-09-25

**Authors:** Zahid Muneer, Christoph Wiesinger, Till Voigtländer, Hauke B. Werner, Johannes Berger, Sonja Forss-Petter

**Affiliations:** 1 Center for Brain Research, Medical University of Vienna, Vienna, Austria; 2 Institute of Neurology, Medical University of Vienna, Vienna, Austria; 3 Department of Neurogenetics, Max Planck Institute of Experimental Medicine, Göttingen, Germany; Clermont Université, France

## Abstract

The inherited peroxisomal disorder X-linked adrenoleukodystrophy (X-ALD), associated with neurodegeneration and inflammatory cerebral demyelination, is caused by mutations in the *ABCD1* gene encoding the peroxisomal ATP-binding cassette (ABC) transporter ABCD1 (ALDP). ABCD1 transports CoA-esters of very long-chain fatty acids (VLCFA) into peroxisomes for degradation by β-oxidation; thus, *ABCD1* deficiency results in VLCFA accumulation. The closest homologue, ABCD2 (ALDRP), when overexpressed, compensates for *ABCD1* deficiency in X-ALD fibroblasts and in *Abcd1*-deficient mice. Microglia/macrophages have emerged as important players in the progression of neuroinflammation. Human monocytes, lacking significant expression of ABCD2, display severely impaired VLCFA metabolism in X-ALD. Here, we used thioglycollate-elicited primary mouse peritoneal macrophages (MPMΦ) from *Abcd1* and *Abcd2* single- and double-deficient mice to establish how these mutations affect VLCFA metabolism. By quantitative RT-PCR, *Abcd2* mRNA was about half as abundant as *Abcd1* mRNA in wild-type and similarly abundant in *Abcd1*-deficient MPMΦ. VLCFA (C26∶0) accumulated about twofold in *Abcd1*-deficient MPMΦ compared with wild-type controls, as measured by gas chromatography-mass spectrometry. In *Abcd2*-deficient macrophages VLCFA levels were normal. However, upon *Abcd1*/*Abcd2* double-deficiency, VLCFA accumulation was markedly increased (sixfold) compared with *Abcd1*-deficient MPMΦ. *Elovl1* mRNA, encoding the rate-limiting enzyme for elongation of VLCFA, was equally abundant across all genotypes. Peroxisomal β-oxidation of C26∶0 amounted to 62% of wild-type activity in *Abcd1*-deficient MPMΦ and was significantly more impaired (29% residual activity) upon *Abcd1*/*Abcd2* double-deficiency. Single *Abcd2* deficiency did not significantly compromise β-oxidation of C26∶0. Thus, the striking accumulation of VLCFA in double-deficient MPMΦ compared with single *Abcd1* deficiency was due to the loss of ABCD2-mediated, compensatory transport of VLCFA into peroxisomes. We propose that moderate endogenous expression of *Abcd2* in *Abcd1*-deficient murine macrophages prevents the severe metabolic phenotype observed in human X-ALD monocytes, which lack appreciable expression of *ABCD2*. This supports upregulation of *ABCD2* as a therapeutic concept in X-ALD.

## Introduction

X-linked adrenoleukodystrophy (X-ALD, OMIM #300100) is an inherited, neurodegenerative disease with variable clinical presentation. Childhood cerebral ALD (CCALD) with rapidly progressive cerebral inflammation and demyelination and adulthood adrenomyeloneuropathy (AMN), characterized by slowly progressive spastic paraparesis, are the two most prominent clinical phenotypes of X-ALD in males [1]–[3]. In heterozygous female carriers X-ALD manifests as a milder variant with myelopathy and/or neuropathy [4], while homozygous females are extremely rare. All forms of X-ALD are caused by mutations in the ATP-binding cassette transporter sub-family D (ALD) member 1 (*ABCD1*) gene. The encoded protein, ABCD1 (formerly adrenoleukodystrophy protein, ALDP), constitutes a half-transporter in the peroxisomal membrane [5]. ABCD1 mediates the transport of CoA-activated very long-chain fatty acids (VLCFA; carbon chain ≥22 C) into peroxisomes, where they are degraded by β-oxidation [6], [7]. Accumulation of saturated VLCFA, in particular C26∶0, in the plasma and tissues of human X-ALD patients is a characteristic biochemical and diagnostic feature of the disease [8].

The endogenous biosynthesis of VLCFA by elongation of long-chain fatty acids (LCFA) accounts for the major pool of VLCFA in human X-ALD patients [9], [10] with only a minor contribution from dietary sources [11]. The enzyme family, elongation of very long-chain fatty acids (ELOVL), mediates the substrate-selective step of the elongation pathway [12]. ELOVL1 was identified to catalyze the synthesis of both saturated (C26∶0) and monounsaturated (C26∶1) VLCFA in human fibroblasts [13].

The capacity for peroxisomal β-oxidation of saturated VLCFA is reduced in patients with X-ALD [14] as well as in *Abcd1*-deficient mice [15]–[17]. In human fibroblasts, *ABCD1* deficiency leads to impaired transport of CoA-esters of VLCFA across the peroxisomal membrane [7]. As a result, the majority of VLCFA escapes degradation via peroxisomal β-oxidation and accumulates in X-ALD conditions. Moreover, the accumulating VLCFA can serve as substrates for ELOVL1, which promotes further increase in chain length and amount of VLCFA culminating with peak levels of C26∶0 [13], [18]. The excess of VLCFA leads to disturbances of intracellular metabolism to varying extent depending on the cell type. Such perturbations may result in alterations of membrane structure, stability and function [19]–[21], oxidative stress [22], [23], energy metabolism [24], [25] and increased expression of inducible nitric oxide synthase [26], [27].

Another peroxisomal ATP-binding cassette (ABC) transporter, ABCD2 (formerly adrenoleukodystrophy-related protein, ALDRP), encoded by the *ABCD2* gene, is the closest homologue of ABCD1 [28]. Overexpression of ABCD2 has been shown to functionally compensate for the impairment in peroxisomal β-oxidation and VLCFA accumulation resulting from *ABCD1* deficiency in human X-ALD fibroblasts and in *Abcd1*-deficient mice [29]–[31]. Furthermore, expression of the human ABC transporters in a heterologous yeast system indicated different but overlapping substrate specificities for ABCD1 and ABCD2 [6], [32]. However, little is known about the endogenous capacity for cross-compensation between ABCD1 and ABCD2 under conditions of insufficiency *in vivo*. In *Abcd2*-deficient mice, saturated VLCFA do not generally accumulate; increased C26∶0 levels have been detected exclusively in the dorsal root ganglia of young *Abcd2*-deficient mice [33]. Instead, accumulation of some saturated LCFA and monounsaturated fatty acids; in particular C20∶0 and ω9-monounsaturated fatty acids was reported in several tissues of *Abcd2*-deficient mice including liver, adrenal gland and sciatic nerve. In other tissues, like spinal cord, the abundance of these fatty acids was normal; and in *Abcd1/Abcd2*-double deficient mice only the level of C22∶1ω9 was elevated compared with *Abcd1* and *Abcd2* single deficient mice [34].

Currently, there is no curative therapy available for most X-ALD patients [35]. Allogeneic bone marrow transplantation or hematopoietic stem cell transplantation [36], [37] and also gene therapy of autologous CD34^+^ hematopoietic stem cells, providing intact ABCD1, can arrest the inflammatory cerebral demyelination in patients at early stages of cerebral ALD [38]. The beneficial effects observed upon transplantation and gene therapy of CD34^+^ hematopoietic stem cells suggest an involvement of the bone marrow-derived macrophages and microglia in this process [39].

In human monocytes/macrophages, *ABCD2* is virtually not expressed [40] and, therefore, cannot compensate for the functional loss of ABCD1 resulting in a severe metabolic phenotype in this cell lineage of X-ALD patients [41]. Interestingly, in the perilesional white matter surrounding demyelinating brain lesions in cerebral ALD, microglia, the resident macrophages of the brain, are particularly vulnerable to neurotoxicity and apoptosis [42], as reviewed in [43]. Furthermore, in X-ALD, VLCFA were found to be enriched in the lysophosphatidylcholine (LPC) fraction [44]; and C24∶0-containing LPC elicited microglial activation and apoptosis when injected into the cerebral cortex of mice [42]. In comparison to other sources of murine macrophages or microglia, large numbers of primary macrophages can be obtained from the peritoneal cavity after stimulation with thioglycollate [45], [46], permitting biochemical studies of VLCFA metabolism in primary macrophages of targeted mouse mutants. These preparations are highly enriched for macrophages and after adherence to plastic culture dishes, most of the cells are positive for the macrophage markers F4/80 or CD11b. In addition to macrophages, some eosinophils, which also express these surface markers, may be present [47]. The vast majority of thioglycollate-elicited primary mouse peritoneal macrophages (MPMФ) are derived from monocytes that are recruited into the peritoneum and partially activated by the inflammatory stimulus. These differ in size, surface markers and function from the residential peritoneal macrophages but show similar phagocytic activity [48]. Genome-wide transcriptome analysis indicates that thioglycollate-induced macrophages are closely related to microglial cells as well as to bone marrow derived macrophages [49].

Here, we used MPMΦ to study the contribution of endogenous *Abcd2* expression to VLCFA metabolism in the macrophage-lineage under *Abcd1* deficiency. We determined the mRNA levels for the peroxisomal fatty acid transporters ABCD1 and ABCD2, as well as for the fatty acyl chain-elongating enzyme ELOVL1, in primary macrophages from wild-type and mutant mice with single or combined *Abcd1* and *Abcd2* deficiencies. Next, we established how these expression patterns correlate with the extent of accumulation and the capacity for degradation of VLCFA. Our results indicate a strong compensatory effect of ABCD2 on the metabolic phenotype of *Abcd1*-deficient murine macrophages.

## Materials and Methods

### Ethics Statement

The care, handling and experiments involving mice were carried out in accordance with the national (Austrian) regulations (BGBl. II Nr. 522/2012) and the directive 2010/63/EU of the European Parliament and the council of the European Union. All procedures were reviewed and approved by the local Animal Care and Use Committee of the Medical University of Vienna and by the Austrian Ministry for Science and Research (BMWF-66.009/0100-II/3b/2013). Mice were euthanized by CO_2_ inhalation before isolation of peritoneal macrophages.

### Animals

Mice with a targeted inactivation (knock-out) of the *Abcd1* gene (B6.129-Abcd1*^tm1Kan^*) have been described previously [15]. Mice with a *null* mutation in the *Abcd2* gene (B6.129-Abcd2*^tm1Sfp^*) were generated as described below. Both mutations had been backcrossed onto the C57BL/6J background for at least twelve generations, before cross-breeding to generate *Abcd1* and *Abcd2* single- and double-deficient mutants and wild-type littermates for this study. Mice of the different allelic combinations appeared at the expected frequencies, were viable and developed on schedule without any overt phenotypical abnormalities. Adult (3 to 6-months-old) male mice were used for isolation of primary peritoneal macrophages. The genotype was determined at weaning and confirmed at sacrifice of experimental animals by standard PCR analysis using the following primers: for ***Abcd1***, forward 5′-TGTCGGGCGTAGACGCTGTCGT-3′ in combination with reverse 5′-CAGGACCACAGCTGTGCGCTTC-3′ for the wild-type allele (yielding a 597-bp PCR product) and reverse 5′-GCCTTCTATCGCCTTCTTGACGAG-3′ for the knock-out (*neomycin resistance* gene, *neo*) allele (yielding a 210-bp PCR product); and for ***Abcd2***, forward 5′-TTCTAAGTGCCGCTGAGCATGC-3′ in combination with reverse 5′-CTGCTGCATTTAGCATGTGTATC-3′ for the wild-type allele (yielding a 466-bp PCR product) and reverse 5′-CCATCTTGTTCAATGGCCGATC-3′ for the knock-out (*neo*) allele (yielding a 322-bp PCR product). All mice for this study were bred and maintained at the local animal facility of the Medical University of Vienna. Mice were housed under standard conditions on a 12/12 h light/dark cycle in a temperature and humidity–controlled environment with standard mouse chow diet and water *ad libitum*.

### Construction of the *Abcd2* targeting vector

**Figure 1:**
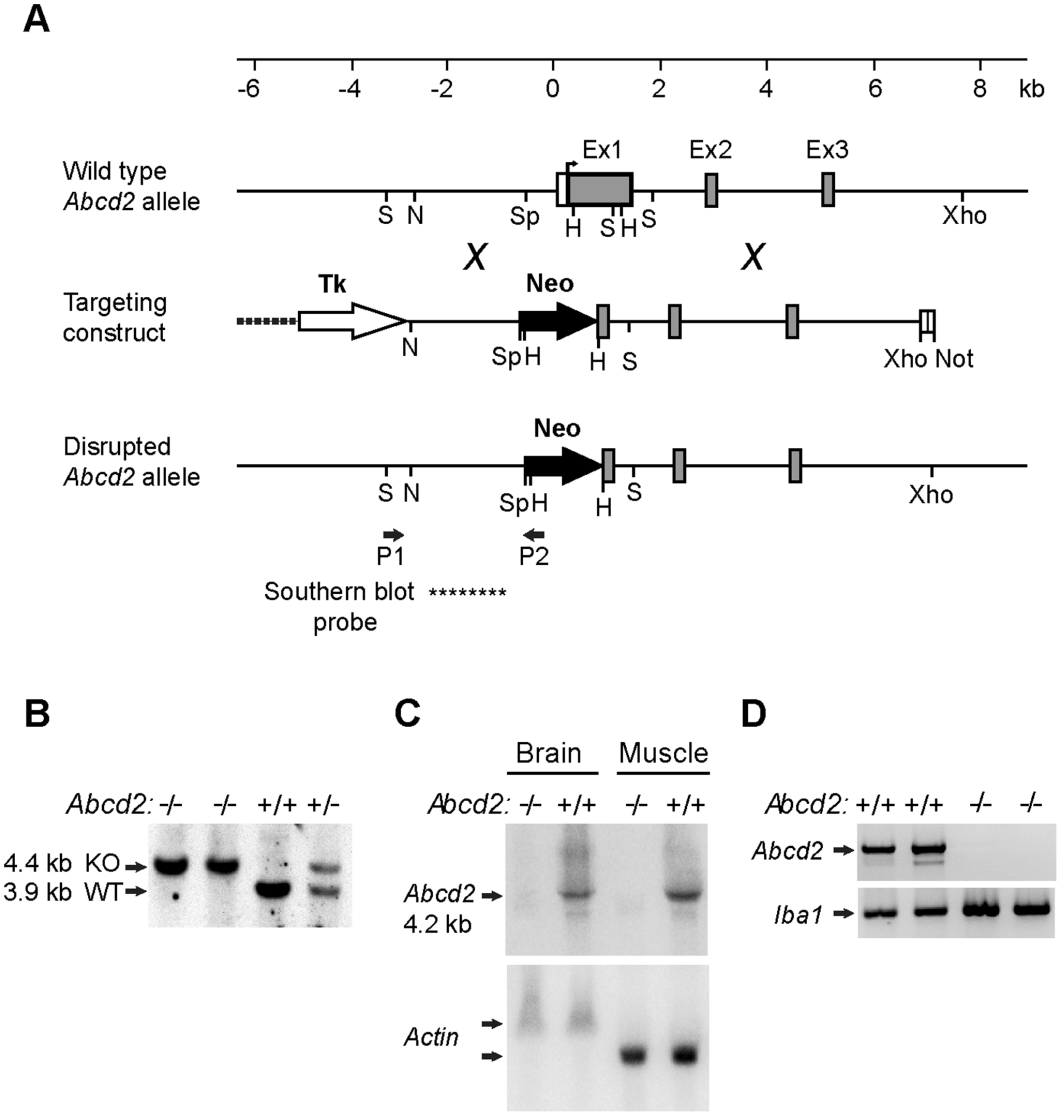
Strategy and molecular evidence for targeted inactivation of the *Abcd2* gene. (A) Structure of the 5′ region of the *Abcd2* gene, the targeting construct and the disrupted gene. Exons (Ex) 1–3 are depicted as boxes with the protein coding region in gray. After homologous recombination, 1.24 kb of the *Abcd2* gene, including the transcription and translation start sites and most of exon 1, is replaced by the *neomycin resistance* gene cassette (Neo) of the targeting construct. Positions of the PCR primers (P1, P2) and the Southern blot probe (****) used in genotyping to detect homologous recombination are indicated. Restriction sites used for cloning or analysis are marked: H, *Hind*III; N, *Nde*I; Not, *Not*I; S, *Sca*I; Sp, *Sph*I and X, *Xho*I. (B) Southern blot analysis of *Sca*I-digested DNA from *Abcd2* ^−/−^, *Abcd2* ^+/+^ and *Abcd2* ^+/−^ mice. Chemiluminescent detection of a digoxygenin-labelled probe from the 5′ flanking DNA verified homologous recombination based on deletion of a *Sca*I-site in exon 1 of *Abcd2* resulting in 3.9-kb and 4.4-kb fragments from the wild-type (WT) and knock-out (KO) alleles, respectively. (C) Northern blot analysis of mRNA from brain and skeletal muscle of adult *Abcd2* ^−/−^ and *Abcd2* ^+/+^ mice. A ^32^P-labelled *Abcd2* cDNA probe hybridized to the major 4.2 kb *Abcd2* mRNA and minor (5.5 and 2.8 kb) variants in both tissues of wild-type mice, but not in *Abcd2* ^−/−^ mice. As a loading control, the blot was re-probed with β-actin cDNA, which in skeletal muscle detects a shorter, more abundant mRNA than in brain. (D) Reverse transcription-coupled PCR analysis of *Abcd2* mRNA expression in primary peritoneal macrophages from two *Abcd2* ^+/+^ and two *Abcd2* ^−/−^ mice. *Abcd2*-specific primers amplified the expected 1,051-bp product in *Abcd2* ^+/+^ but not in *Abcd2* ^−/−^ macrophages. As a positive control, amplification of *Iba1* cDNA (310-bp fragment) was confirmed in all samples.

A mouse 129/SvJ genomic library (λFIXII; Stratagene) was screened with a probe covering nucleotides 16−1,078 of the murine *Abcd2* cDNA (GenBank™ Accession No. NM 011994). From a phage λ-clone containing 17 kb of the *Abcd2* gene, including exon 1 through intron 3 and 3.5 kb of 5′-flanking DNA, genomic DNA fragments were subcloned into plasmids to generate a gene-replacement vector for positive-negative selection [50]. The final targeting construct (Fig. 1A) contained in 5′ to 3′ order: a 2.2-kb *Xho*I−*Nde*I fragment carrying the *thymidine kinase* gene from pMC1TK (Stratagene); a 2.1-kb *Nde*I− *Sph*I fragment (−2.5 kb to −422 relative to the translation start site of *Abcd2*) representing the 5′ (short) *Abcd2* homology region, adapted with a 3′ *Hind*III site; a 1.15-kb *neo* gene cassette from pMCneopA (Stratagene) adapted with 3′ *Hind*III sites; and a 6-kb *Hind*III−*Not*I fragment accommodating the 3′ (long) *Abcd2* homology region [from *Hind*III (+816) in exon 1 to *Xho*I in intron 3, cloned into a polylinker with a *Not*I site] inserted between *Sal*I and *Not*I of the pBluescript KSII (Stratagene) vector backbone. Thus, the *neo* cassette replaces a 1,238-bp region of the *Abcd2* gene, between *Sph*I (−422) and *Hind*III (+816), which contains the promoter and most of exon 1. Both *neo* and *thymidine kinase* were inserted in sense orientation relative to *Abcd2* (Fig. 1A).

### Generation of *Abcd2*-deficient mice

Mouse RI embryonic stem (ES) cells [51], kindly provided by A. Nagy (Toronto), were transfected by electroporation using 50 µg of the *Not*I-linearized targeting vector. Colonies were isolated after G418/gancyclovir selection as previously described [50], [52]. Double-resistant clones resulting from homologous recombination were identified by PCR amplification of a 3.1-kb genomic *Abcd2-neo* junction fragment from DNA of heat-lysed cells using *Abcd2* forward primer (5′-AGGATCTGCTTAAGAGTTCCACT-3′) at position P1, flanking the homology region, and *neo* reverse primer (5′-CCATCTTGTTCAATGGCCGATC-3′) at position P2 (as indicated in Fig. 1A). The initial denaturation at 94°C for 3 min was followed by 32 cycles of PCR (denaturation at 94°C for 30 s, annealing at 61°C for 30 s, and extension at 72°C for 2 min) and final extension at 72°C for 2 min. Individual PCR-positive clones were expanded and homologous recombination was confirmed by Southern blot analysis of *Sca*I-digested DNA (8 µg/sample) using a digoxygenin-labelled hybridization probe (indicated in Fig. 1A) derived from the 5′-flanking region, upstream from the *neo* insertion.

Microinjection of selected ES cells into C57BL/6J blastocysts and embryo transfer to foster mothers were performed by standard procedures. Resulting chimeras were mated with C57BL/6J mice and germ line-transmission was obtained from one founder. Heterozygous (*Abcd2^+/−^*) F1 mice were again crossed with C57BL/6J mice and F2 heterozygous mutants were interbred to generate homozygous (*Abcd2^−/−^*) mice in the F3 generation. The *Abcd2* genotype of these mice was determined by allele-specific PCR and Southern blot analyses using genomic DNA (Fig. 1B) as outlined for ES cells.

### RNA analyses for verification of functional disruption of *Abcd2*

Northern blot analysis was used to confirm the absence of functional *Abcd2* gene expression in *Abcd2^−/−^* mutant mice. Total RNA was isolated from flash frozen tissues [53] of 2.5-months-old *Abcd2* ^+/+^ and *Abcd2* ^−/−^ littermates. For each sample, the polyA^+^-RNA fraction (approximately 2 µg) obtained by oligo(dT) selection of 100 µg total RNA was size-separated on formaldehyde-agarose gels to prepare northern blots as previously described [54]. A gene-specific, [α-^32^P]dCTP-labelled hybridization probe covering exon 1 was generated by random priming with *Abcd2* cDNA as a template. As a control of loading and transfer, the blot was stripped and reprobed with β-actin cDNA. All blots were washed to high stringency (at 68°C in 0.2 x SSC, 0.2% SDS) and monitored with a phoshorimager (Molecular Imager FX System, Bio-Rad).

For end point RT-PCR analysis of *Abcd2* mRNA expression in MPMФ, total cellular RNA was isolated and reverse transcribed as described for the qRT-PCR analysis (see below). Diluted cDNA, corresponding to 4 ng of total RNA, was applied for 35 cycles of PCR using GoTaq polymerase (Promega) with *Abcd2* forward primer (5′-GAAGCCTCGGACTTTCATCATC-3′) and reverse primer (5′-GTGTAATTATGGGAACATTTTCAC-3′) from exon 1 and exon 5, respectively, at an annealing temperature of 59°C, to generate a 1,051-bp product in *Abcd2^+/+^* cDNA samples. No amplicon was observed when reverse transcriptase was omitted or when genomic DNA was used as template. As a positive control, the macrophage marker ionized calcium-binding adapter molecule 1 (*Iba1*), also known as allograft inflammatory factor 1 (*Aif1*), was amplified from the same cDNA samples under identical conditions, except that annealing was at 54°C, with *Iba1* forward primer (5′-AGAAGAGACTGGGGAGCTGGT-3′) from exon 3 and reverse primer (5′-CCAAGTTTCTCCAGCATTCGC -3′) from exon 7 (based on GenBank™ Accession No. NM 019467.2) generating a specific 310-bp PCR product.

### Isolation and Cell Culture of Mouse Peritoneal Macrophages

Primary mouse peritoneal macrophages (MPMΦ) were isolated from wild-type and knock-out mice as described in [55] with some minor modifications. Briefly, mice were injected intraperitoneally with 2 ml of 4% thioglycollate medium (Sigma). Four days later, mice were sacrificed for peritoneal lavage with 10 ml of complete medium [RMPI-1640 medium (PAA) supplemented with 10% fetal bovine serum (PAA), 100 units/ml each of penicillin and streptomycin (Lonza) and 2.5 µg/ml Fungizone (Gibco)] using a syringe with a 23G needle. The recovered cells were centrifuged at 300 × *g* at 4°C for 10 min; the supernatant was aspirated and the cell pellet resuspended and washed twice in 10 ml complete RPMI-1640 medium. Finally, cells were counted and the required numbers were plated and cultured at 37°C with 5% CO_2_ for 4 h, during which the MPMΦ attach to the cell culture plate (CELLSTAR, Greiner Bio-One) while the vast majority of other cell types from the peritoneal lavage remain in suspension and are removed by aspiration. After this incubation period, the attached MPMΦ were washed with phosphate buffered saline (PBS) and used according to the experimental requirements. Unless stated otherwise, the cells were maintained for 20 h in complete medium at 37°C with 5% CO_2_ before harvesting.

### RNA isolation and quantitative reverse transcription-coupled PCR (qRT-PCR)

MPMΦ (3×10^5^ cells/well) were seeded in 6-well cell culture plates. At harvest, the attached MPMΦ were washed with PBS and total RNA was isolated using the RNeasy Mini kit (Qiagen), including on-column DNA digestion with RNase-free DNase (Qiagen), according to the manufacturer’s instructions. RNA concentrations were determined using a NanoDrop spectrophotometer (PEQlab). Total RNA (80 ng) was reverse transcribed into cDNA in 20 µl final volume using the iScript™ cDNA synthesis kit (Bio-Rad) at 25°C for 5 min, 42°C for 30 min and 85°C for 5 min and holding at 4°C. The cDNA was diluted 1∶5 and 5-µl aliquots were applied for two-step qRT-PCR (denaturation at 95°C, annealing/extension at 60°C) using SsoFast EvaGreen Supermix (Bio-Rad) and the CFX96 Real-Time PCR Detection System (Bio-Rad) according to the manufacturer’s recommendations together with the following gene specific primer combinations: ***Hprt***, forward 5′-ACTTCAGGGATTTGAATCACGTT-3′ and reverse 5′-GCAGATGGCCACAGGACTAGA-3′, product size 154 bp; ***Abcd1***, forward 5′-GCTGTGACCTCCTACACTCTCC-3′ and reverse 5′-AGTAGTGCCAGTTCCACCTCA-3′, product size 251 bp; ***Abcd2***, forward 5′-GAACTACCCCTCAGCGACAC-3′ and reverse 5′- ATGGCCTCTGTGGAATATAGAAC-3′, product size 280 bp; and ***Elovl1***, forward 5′-ATTGAGCTGATGGACACAGTGAT-3′ and reverse 5′-GACCAGGACAAACTGGATCAGC-3′, product size 279 bp.

The absolute quantification of each mRNA was based on standard curves, which were generated from serial dilutions of known copy numbers of linearized plasmids containing the corresponding cDNA region of murine *Abcd1, Abcd2, Elovl1* or *Hprt*. The quantity obtained for each mRNA after reverse transcription to cDNA (Table S1 and Table S2) was normalized to that of *Hprt* in order to compensate for variation in amount of total RNA and/or cDNA conversion between samples.

### VLCFA measurements

Deuterium-labelled VLCFA standards: [3,3,5,5-^2^H_4_]-hexacosanoic acid (^2^H_4_-C26∶0), [3,3,5,5-^2^H_4_]-tetracosanoic acid (^2^H_4_-C24∶0) and [3,3,5,5-^2^H_4_]-docosanoic acid (^2^H_4_-C22∶0) were obtained from Dr. Herman J. ten Brink (Free University Hospital, Amsterdam, The Netherlands); and [7,7,8,8-^2^H_4_]-palmitic acid (^2^H_4_-C16∶0) was from Cambridge Isotope Laboratories Inc. (Andover, MA, USA); and the unlabelled fatty acids C26∶0, C24∶0, C22∶0, and C16∶0 were from Sigma-Aldrich.

For each sample, 5−7×10^6^ peritoneal cells were seeded in one culture plate and maintained in complete medium for either 1 day or 5 days. At harvest, the MPMΦ were washed three times with PBS and pelleted. Cell pellets were suspended in distilled water and sonicated three times for 30 s on ice. Protein concentration was determined by the method of Lowry using bovine serum albumin (Sigma-Aldrich) as standard [56]. Extraction and quantitative analysis of fatty acids by gas chromatography-mass spectrometry (GC-MS) were carried out as previously described [57]. GC-MS measurements were carried out in a TRACE MS Plus gas chromatograph single quadrupol mass spectrometer (Thermo Fisher Scientific) equipped with a J&W Scientific DB-1ms capillary column (30 m x 0.25 mm I.D., film thickness 0.25 µm; Agilent Technologies). Data were analyzed with the Finnigan Xcalibur™ software package (Thermo Fisher Scientific) using calibration curves obtained from unlabelled fatty acids in the concentration range from 0.005 to 0.2 µg/ml for C26∶0, from 0.25 to 2.5 µg/ml for C22∶0 and C24∶0, and from 5 to 100 µg/ml for C16∶0.

### β-Oxidation of 1-^14^C-labelled fatty acids

The MPMΦ obtained after selective adherence of 2×10^6^ peritoneal cells, seeded in one culture plate and maintained in complete medium for 20 h, were washed with PBS before harvesting and resuspending in 200 µl of buffer (250 mM sucrose, 10 mM Tris-Cl, pH 8.0). The protein concentration was measured in an aliquot of each sample using the CBQCA Protein Quantitation Kit (Molecular Probes). To determine the β-oxidation activity for C26∶0 and C16∶0, aliquots of 100 µl and 33 µl were used, respectively.

Radiolabelled 1-^14^C palmitic acid (C16∶0; ARC 0172A) and 1-^14^C hexacosanoic acid (C26∶0; ARC 1253) were obtained from American Radiolabeled Chemicals. Free fatty acids in ethanol were aliquoted into glass reaction tubes, dried under a stream of nitrogen and solubilized in 10 mg/ml α-cyclodextrin by ultrasonication. β-Oxidation of labelled fatty acids to acetate was carried out according to [58] with modifications as described previously [7]. Briefly, for each sample the reaction mix of 250 µl contained 4 µM radiolabelled fatty acid (either C16∶0 or C26∶0), 2 mg/ml α-cyclodextrin, 250 mM sucrose, 30 mM KCl, 20 mM Tris-Cl (pH 8.0), 8.5 mM ATP, 8.5 mM MgCl_2_, 2.5 mM l-carnitine, 1 mM DTT, 1 mM NAD^+^, 0.5 mM malate, 0.2 mM EDTA, 0.17 mM FAD, 0.16 mM CoA and cell preparation (as described above). Reactions were started by addition of cellular protein, incubated for 1 h at 37°C and stopped by adding KOH and heating at 60°C for 1 h. After protein precipitation by HClO_4_, a Folch partition was carried out and the amount of ^14^C-acetate in the aqueous phase was determined in a scintillation counter.

### Statistical analyses

Statistical analyses were carried out either with two-tailed t-test or one-way analysis of variance (ANOVA) with appropriate post-hoc test. Statistical analyses for VLCFA were performed on log-transformed data using one-way ANOVA followed by Tukey’s post-hoc test. Thereafter, these data were back-transformed to obtain and represent the geometric means with error bars showing the asymmetrical standard deviations. Statistical analyses for β-oxidation assays were performed using one-way ANOVA followed by Tukey’s post-hoc test. Data from β-oxidation assays and qRT-PCR are represented as arithmetic means ± standard deviation (SD). Differences in mean values were considered statistically significant at p<0.05.

## Results

### Targeted inactivation of the murine *Abcd2* gene

To study the compensatory role of *Abcd2* in tissues and distinct cell types of X-ALD mice, we generated mice with a null mutation in the *Abcd2* gene by applying a similar strategy as previously used for the targeted inactivation of *Abcd1* [15]. Briefly, the targeting vector for homologous recombination in mouse ES cells was constructed from isogenic (129Sv) DNA encompassing the 5′ region of the murine *Abcd2* gene (Fig. 1A). Between the “short arm” (3.5 kb of DNA upstream from exon 1) and the “long arm” (6 kb from the 3′ end of exon 1 through intron 3) of the targeting vector, 1,238 bp of the *Abcd2* gene containing 250 bp of the promoter region and most of exon 1 were replaced by a *neo* gene cassette, which also served as a marker for positive selection. This deletion eliminates the transcription and translation start sites and the coding capacity for the 272 amino-terminal amino acids of ABCD2 [59]. A *thymidine kinase* gene cassette was inserted 5′ to the homology region for negative selection against random integration events. ES cells were transfected with the targeting vector and double (G418, gancyclovir)-resistant recombinant clones selected, of which 10% were identified by PCR to result from homologous recombination. Correct gene targeting was confirmed by Southern blot analysis discriminating between wild-type (*Abcd2* ^+^) and disrupted (*Abcd2* ^−^) alleles based on the loss of a diagnostic *Sca*I restriction site in exon 1 (Fig. 1A).

Germline chimeras were obtained from a correctly targeted ES clone injected into C57BL/6 blastocysts. The progeny obtained after breeding of this founder and subsequent generations with C57BL/6J mice gave rise to a permanent mouse line (B6.129-Abcd2*^tm1Sfp^*) carrying the disrupted *Abcd2* gene (here referred to as *Abcd2*-deficient, *Abcd2* ^−/−^, or *Abcd2* knock-out). The genotype was initially determined by PCR (data not shown) and Southern blot analysis (Fig. 1B) to confirm the gene structure expected after a unique homologous recombination event. Heterozygous and homozygous animals were obtained at the expected Mendelian ratios and were viable, fertile and developed into adults with normal home cage behaviour and healthy appearance. Starting around 12 months of age, *Abcd2* ^−/−^ mice developed a sensory–motor impairment (data not shown), which has been reported previously in an independent *Abcd2*-deficient mouse strain [31], [33]. This ataxic phenotype developed with a similar age-of-onset also in *Abcd1*/*Abcd2* double-deficient mice.

### Loss of *Abcd2* mRNA expression in gene targeted mice

To verify that homologous recombination had inactivated functional expression of *Abcd2*, we assessed the presence of *Abcd2* mRNA by Northern blot analysis in brain and skeletal muscle, two tissues in which *Abcd2* is normally abundantly expressed [60]. The *Abcd2*-specific cDNA probe detected the major 4.2 kb *Abcd2* mRNA, as well as the minor 5.5 and 2.8 kb transcripts arising from alternative polyadenylation sites, in both tissues of wild-type but not *Abcd2* ^−/−^ mice (Fig. 1C). The loading control, β-actin, gave similar signals in corresponding wild-type and knock-out tissues.

The absence of *Abcd2* mRNA was also verified in primary mouse peritoneal macrophages (MPMФ) isolated from *Abcd2^−/−^* mice by end point RT-PCR using total cellular RNA. In wild-type cells, RT-PCR with *Abcd2*-specific primers robustly amplified the expected product, whereas no signal was obtained in *Abcd2* ^−/−^ MPMФ (Fig. 1D). As a positive control for the quality of the RNA/cDNA preparations, the macrophage marker *Iba1* could be easily amplified in all cDNA samples. Taken together, these expression studies confirm the absence of functional *Abcd2* mRNA in cells and tissues of the *Abcd2* knock-out mice, validating that the gene disruption generated a *null* mutation.

By cross-breeding the *Abcd1*- and *Abcd2*-deficient mutant strains, we generated mice with single and combined *Abcd1*/*Abcd2*-deficiencies and appropriate wild-type littermate controls, all on the C57BL/6J background. Mice of the different allelic combinations appeared at the expected frequencies, were viable and reached adulthood without overt phenotypical abnormalities. In the present study only young male mice were used as donors of thioglycollate-elicited peritoneal macrophages, because in X-linked ALD, predominantly the male (hemizygous) patients are severely affected.

### *Abcd2* mRNA is expressed at half the level of *Abcd1* mRNA in mouse peritoneal macrophages

**Figure 2:**
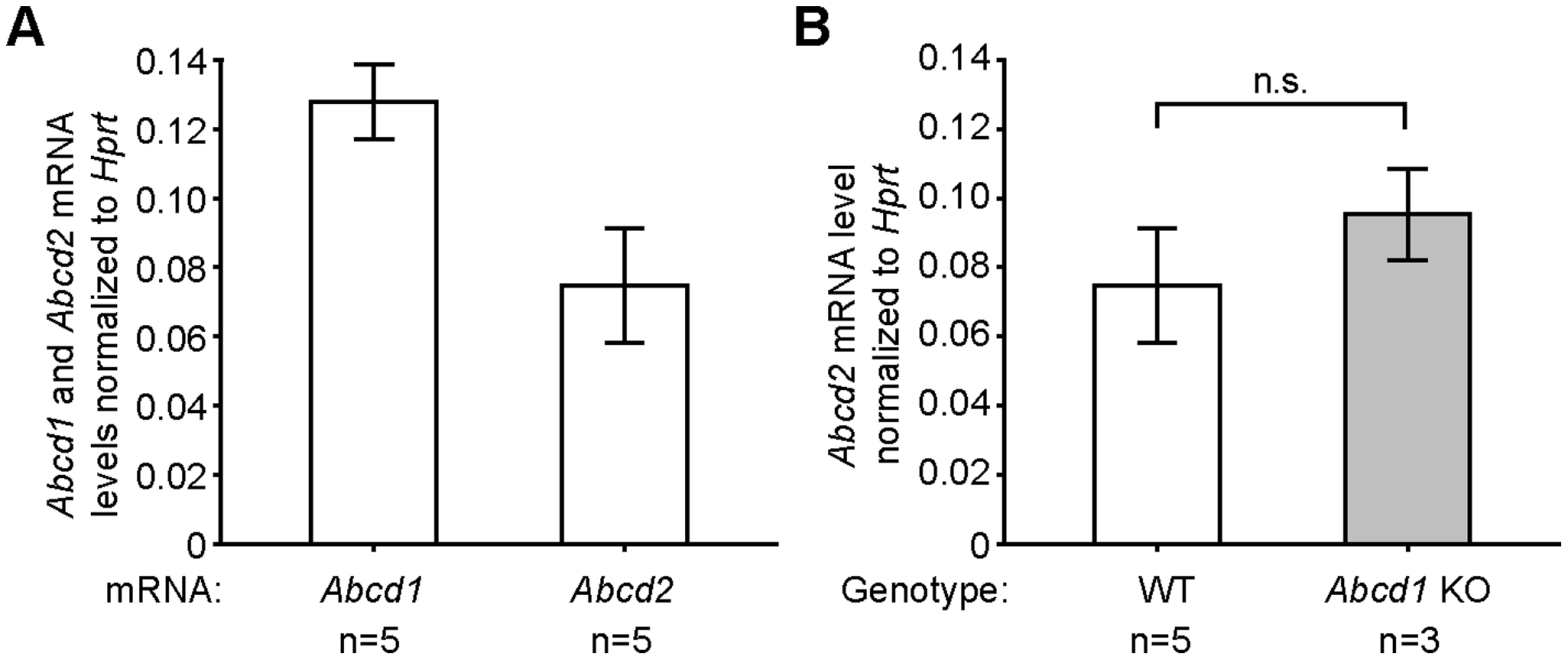
Endogenous *Abcd1* and *Abcd2* mRNA levels in peritoneal macrophages of wild-type and *Abcd1*-deficient mice. (A) The *Abcd1* and *Abcd2* mRNA copy numbers in C57BL/6J wild-type MPMΦ were determined by qRT-PCR. (B) Comparison of *Abcd2* mRNA levels in wild-type (WT) and *Abcd1*-deficient (KO) MPMΦ. The numbers of samples (*n*) are indicated below the graphs. The graphs indicate mean values ± SD after normalization to the level of *Hprt* mRNA in each sample; *n.s*., no statistically significant difference.

First, we explored the basal expression of the *Abcd1* and *Abcd2* genes in MPMΦ collected from wild-type mice and then compared these with the levels in *Abcd1* and *Abcd2*-deficient MPMΦ. The absolute quantity of each mRNA was determined by qRT-PCR analysis and normalized to the level of *Hprt* mRNA (Fig. 2A and Table S1). In wild-type cells, both mRNAs were detected at low abundance; however, the *Abcd2* mRNA was present at 58% of the level of the *Abcd1* mRNA (Fig. 2A). Because we had previously found *Abcd2* mRNA expression to be altered in hepatic tissue of *Abcd1*-deficient mice [61], we determined the *Abcd2* mRNA levels in *Abcd1*-deficient MPMΦ in order to uncover any potential compensatory dysregulation. However, there was no statistically significant difference between the *Abcd2* mRNA levels of wild-type and *Abcd1*-deficient MPMΦ (Fig. 2B). *Vice versa*, the *Abcd1* mRNA remained at wild-type level in MPMΦ from *Abcd2*-deficient mice (Fig. S1).

### Inactivation of *Abcd2* alone has no impact on VLCFA levels of MPMΦ but a strong synergistic effect in combined *Abcd1*/*Abcd2* deficiency

**Figure 3:**
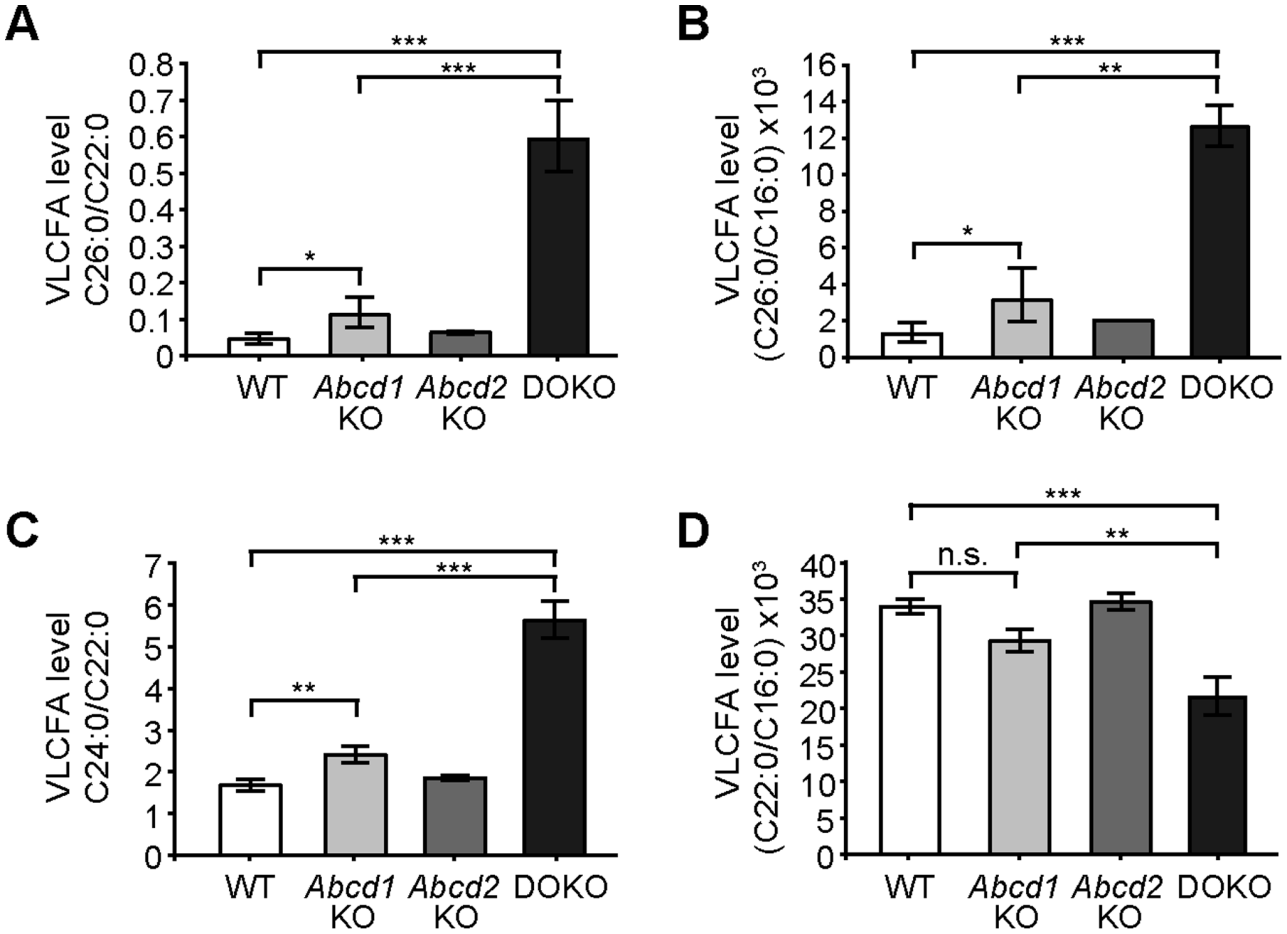
VLCFA levels in wild-type, *Abcd1* and *Abcd2* single-deficient and *Abcd1/Abcd2* double-deficient mouse peritoneal macrophages. The concentrations of the VLCFA species C26∶0, C24∶0 and C22∶0 and the LCFA C16∶0 were determined by GC-MS. The relative amounts of fatty acids, expressed as ratios: (A) C26∶0/C22∶0; (B) C26∶0/C16∶0; (C) C24∶0/C22∶0 and (D), C22∶0/C16∶0 were analyzed in mouse peritoneal macrophages of wild-type (WT), *Abcd1*-deficient (*Abcd1* KO), *Abcd2*-deficient (*Abcd2* KO) and *Abcd1/Abcd2* double-deficient (DOKO) mice after 5 days in culture (*n* = 3). The graphs indicate geometric means ± SD (asymmetrical). Statistically significant differences are indicated: * *p*<0.05, ** *p*<0.01, *** *p*<0.001; *n.s*., no statistically significant difference.

MPMΦ isolated from mice with single or combined *Abcd1* and *Abcd2* deficiency or from wild-type littermates were cultured for either one or five days before harvesting for fatty acid analysis. The amounts of VLCFA (C26∶0, C24∶0, C22∶0) and LCFA (C16∶0) were measured by GC-MS. First, we established the extent of accumulation of saturated VLCFA in *Abcd1*-deficient MPMΦ. Compared with wild-type levels, we found a two-fold increase in the ratio C26∶0/C22∶0 (Fig. 3A). There was no statistically significant difference in the C26∶0/C22∶0 ratio of *Abcd2*-deficient MPMΦ when compared with wild-type. However, we noticed strikingly increased accumulation of C26∶0 in MPMΦ from *Abcd1/Abcd2* double-deficient mice, as judged by the ratio C26∶0/C22∶0 (Fig. 3A) as well as the absolute amounts (Fig. S2). The difference in accumulation (C26∶0/C22∶0) was statistically highly significant in comparison with wild-type or *Abcd2*-deficient or even with *Abcd1*-deficient samples (p<0.001). We observed similar C26∶0/C22∶0 profiles in MPMΦ harvested after five days (Fig. 3A) or after one day (Fig. S3) in cell culture. Because the level of C22∶0 remains unaltered or even drops somewhat in X-ALD, we also established the levels of C16∶0 in all samples, as an additional reference for comparisons of the different VLCFA species, in addition to their absolute values normalized to the amount of protein in the cell homogenate (Fig. S2). The C16∶0 levels were comparable across the different genotypes when normalizing to protein (Fig. S2D). The ratio C26∶0/C16∶0 (Fig. 3B) was increased in MPMΦ from *Abcd1*-deficient mice to a similar extent as that of C26∶0/C22∶0 (Fig. 3A). Also the marked increase in C26∶0 levels of *Abcd1*/*Abcd2* double-deficient compared with *Abcd1*-deficient cells was obvious in the C26∶0/C16∶0 ratios (p<0.01) (Fig. 3B). Whereas the level of C24∶0 was only slightly elevated upon *Abcd1* deficiency, also this fatty acid accumulated substantially in the *Abcd1*/*Abcd2* double-deficient MPMΦ, as indicated by the C24∶0/C22∶0 ratio (Fig. 3C) as well as the absolute values (Fig. S2B). In contrast, the levels of C22∶0 were slightly lower in MPMΦ from *Abcd1-*deficient mice, both when expressed as ratio to C16∶0 (Fig. 3D) or after normalizing to protein amounts (Fig. S2C), but the reduction did not reach statistical significance. Interestingly, there was even a further, statistically significant decrease in the C22∶0/C16∶0 ratios in *Abcd1*/*Abcd2* double-deficient MPMΦ when compared with wild-type, *Abcd1*-deficient and *Abcd2*-deficient MPMΦ (Fig. 3D).

### Elovl1 mRNA levels of MPMΦ are not affected by *Abcd1* or *Abcd2* deficiency

**Figure 4:**
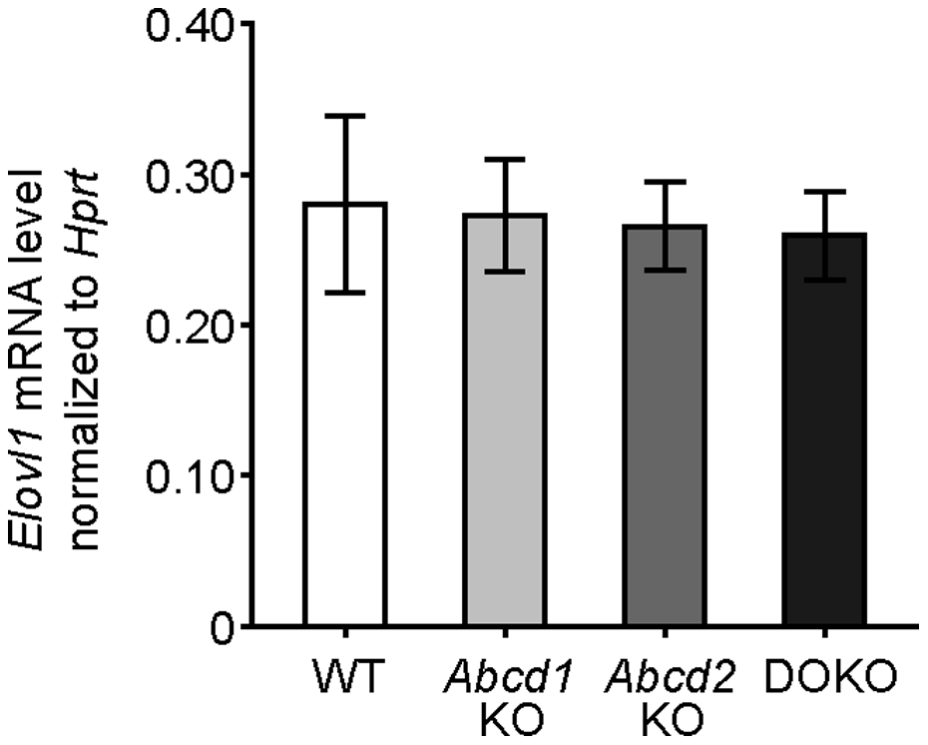
*Elovl1* mRNA levels in wild-type, *Abcd1-, Abcd2-,* and *Abcd1/Abcd2* double-deficient mouse peritoneal macrophages. The *Elovl1* mRNA copy numbers were determined by qRT-PCR in total RNA from mouse peritoneal macrophages of wild-type (WT), *Abcd1*-deficient (*Abcd1* KO), *Abcd2*-deficient (*Abcd2* KO) and *Abcd1/Abcd2* double-deficient (DOKO) mice. The graphs indicate mean values ± SD after normalization to the level of *Hprt* mRNA in each sample (*n* = 3).

ELOVL1 is responsible for the carbon-chain elongation of VLCFA (C22∶0, C24∶0, C26∶0). As the marked increase in C26∶0 accumulation in *Abcd1*/*Abcd2* double-deficient MPMΦ compared with isolated *Abcd1* deficiency could be partially, or entirely, due to increased elongation of VLCFA, we measured the *Elovl1* mRNA levels by qRT-PCR in wild-type and *Abcd1*/*Abcd2* single- and double-deficient MPMΦ. After normalization to *Hprt* mRNA levels, we observed no difference in *Elovl1* mRNA expression in any of the mutant genotypes (Fig. 4 and Table S2). Thus, at least at the level of gene expression, *Elovl1*, encoding the rate limiting enzyme for elongation of VLCFA, was not upregulated.

### Peroxisomal β-oxidation of C26∶0 is markedly more impaired in *Abcd1*/*Abcd2* double-deficient MPMΦ than in *Abcd1* single deficiency

**Figure 5:**
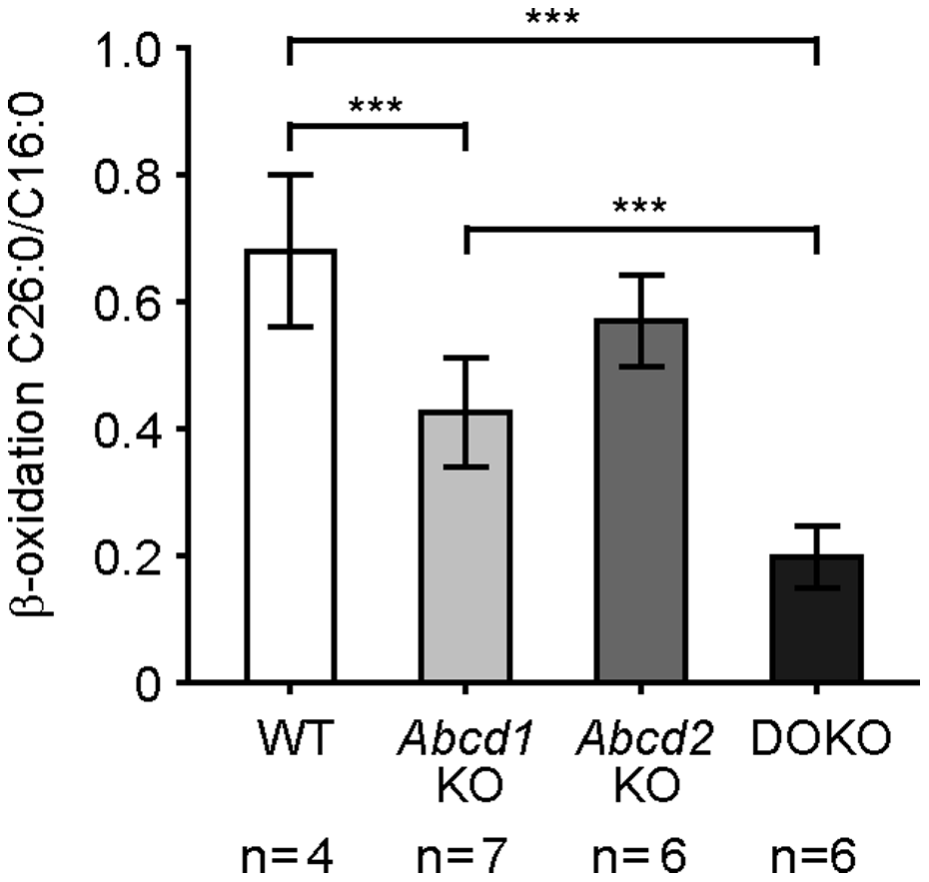
β-Oxidation activity for C26∶0 in wild-type, *Abcd1-, Abcd2-,* and *Abcd1/Abcd2* double-deficient mouse peritoneal macrophages. The rates of β-oxidation for radiolabelled C26∶0 and C16∶0 fatty acid substrates were measured in wild-type (WT), *Abcd1*-deficient (*Abcd1* KO), *Abcd2*-deficient (*Abcd2* KO) and *Abcd1/Abcd2* double-deficient (DOKO) cell preparations. The results are shown as mean values ± SD of the ratio between C26∶0 and C16∶0. The numbers of samples (*n*) are indicated below the graphs. Statistically significant differences are indicated; *** *p*<0.001.

To establish whether the degradation of VLCFA is impaired in the different mutant macrophages, we determined the rate of peroxisomal β-oxidation in MPMΦ from wild-type, *Abcd1*-, *Abcd2*- and *Abcd1*/*Abcd2* double-deficient mice using radiolabelled C26∶0 as substrate. As a control, and for normalization of the overall activity of each cell preparation, we measured the rate of β-oxidation for the LCFA C16∶0, which in mammals is primarily degraded in mitochondria. We found a highly significant (p<0.001) decrease (38%) in the relative β-oxidation activity for C26∶0 (depicted as ratio to C16∶0) in *Abcd1*-deficient MPMΦ compared with wild-type values (Fig. 5). Isolated *Abcd2* deficiency had no statistically significant effect on the capacity for β-oxidation of either substrate. However, there was a highly significant (p<0.001) further reduction in the β-oxidation activity for C26∶0 in *Abcd1*/*Abcd2* double-deficient MPMΦ when compared with *Abcd1*-deficient (54%) as well as wild-type (71%) and *Abcd2*-deficient (65%) MPMΦ (Fig. 5). Similar results were obtained when considering the absolute values of the β-oxidation rates for C26∶0 normalized to the amount of cellular protein in the MPMΦ samples (Fig. S4A). The β-oxidation activity for C16∶0 was similar in MPMΦ of all different genotypes (Fig. S4B).

## Discussion

Our results show that primary macrophages from *Abcd1*/*Abcd2* double-deficient mice have a considerably more severe metabolic phenotype than those with single *Abcd1* deficiency, as documented by a markedly reduced rate of peroxisomal β-oxidation for C26∶0 leading to the strongly increased accumulation of saturated VLCFA (C26∶0 and C24∶0). Inactivation of *Abcd2* alone has no significant effect on the levels of saturated VLCFA in most murine tissues including brain, spinal cord and sciatic nerve, which are affected by the pathology in X-ALD [31], which is also the case in our *Abcd2* ^−/−^ mouse strain (Forss-Petter, unpublished observation). One notable exception are the dorsal root ganglia [33], which may explain the earlier onset of a sensory motor phenotype in *Abcd1/Abcd2* double-deficient and *Abcd2* single-deficient mice compared with *Abcd1*-deficient mice [31]. Considering that ubiquitous transgenic overexpression of the murine ABCD2 protein in *Abcd1*-deficient mice rescued the metabolic impairment and the motor-behavioural phenotype [31], a more dramatic phenotype might have been anticipated in *Abcd1*/*Abcd2* double-mutant mice. However, the additive but not amplified severity of the metabolic and behavioural deficits might be partially explained by the rather complementary expression patterns of these two peroxisomal ABC transporters [41], [60], [62].

In MPMΦ, we found both the *Abcd1* and *Abcd2* mRNAs to be expressed at relatively low abundance (*i.e.* estimated to represent less than 0.01% of the total mRNA molecules and lower than the level of *Hprt* mRNA). By qRT-PCR, the steady-state level of the *Abcd1* mRNA was about twice that of *Abcd2*. Thus, MPMΦ constitute a good model system to evaluate the level of *Abcd2* expression required to prevent a severe metabolic phenotype upon *Abcd1* deficiency. Our results revealed about twofold higher levels of VLCFA in *Abcd1*-deficient MPMΦ, as judged by the ratio C26∶0/C22∶0, in agreement with previous observations [27]. This is similar to the extent of VLCFA storage in modestly affected tissues like the liver of X-ALD mice [63]. Interestingly, in *Abcd1*/*Abcd2* double-deficient MPMΦ, we found an additional sixfold increase in accumulation of C26∶0 when compared with *Abcd1* single-deficiency, which amounted to approximately twelvefold higher than wild-type levels. In brain and adrenal glands, the pathologically affected tissues in human X-ALD patients, the C26∶0/C22∶0 ratio is elevated up to fourfold in *Abcd1*-deficient mice [15], [63]. The extent of VLCFA accumulation in myelin-enriched tissues, such as spinal cord and sciatic nerve, was reported to be five to sixfold in *Abcd1*-deficient mice and was not further elevated in *Abcd1/Abcd2* double-deficient mice [31]. Thus, the twelvefold accumulation of C26∶0 that we observed here in *Abcd1*/*Abcd2* double-deficient MPMΦ is indeed remarkably strong. These results indicate that ABCD2 provides an alternative transport route across the peroxisomal membrane for saturated VLCFA destined for β-oxidation, thereby counteracting a more dramatic accumulation in MPMΦ lacking functional ABCD1 and rendering the levels of VLCFA only modestly higher than in wild-type macrophages.

The increased accumulation of saturated VLCFA in MPMΦ from *Abcd1*/*Abcd2* double-deficient mice most likely results from their strongly decreased capacity (29% of wild-type activity) to catabolize VLCFA via the peroxisomal β-oxidation pathway. Thus, also in murine monocyte-derived macrophages, the primary peroxisomal transporter for saturated VLCFA-CoA is ABCD1. However, in contrast to human monocytes [41] or human fibroblasts [7], in the absence of ABCD1, sufficient amounts of endogenous ABCD2 are present to maintain a substantial β-oxidation capacity for VLCFA substrates (62% of wild-type β-oxidation activity for C26∶0, Fig. 5). The most likely candidate for mediating the residual β-oxidation activity (29%) for C26∶0 in the *Abcd1*/*Abcd2* double-deficient MPMΦ is the third peroxisomal ABC transporter, ABCD3, in similarity to human X-ALD fibroblasts [7] and monocytes [41]. Unsaturated long- branched-chain and dicarboxylic fatty acids were recently shown to be the preferred substrates for human ABCD3 in a heterologous expression system, while low activity was obtained with C26∶0 [64].

Another possibility to promote accumulation of VLCFA would be through increased elongation of fatty acids boosting the biosynthesis of C26∶0. The chain length-specific enzyme in this process is ELOVL1 [12]. In a model based on knock-down of *Abcd1* mRNA in rat B12 oligodendrocytes, elevated *Elovl1* mRNA levels were reported to coincide with increased VLCFA levels [65] indicating an association between VLCFA levels and the regulation of *Elovl1* expression. However, we found no difference from wild-type levels of *Elovl1* mRNA in any of the mutant MPMΦ by qRT-PCR. This indicates that regulation at the level of *Elovl1* gene expression is unlikely to account for the increased accumulation of VLCFA in *Abcd1*/*Abcd2* double-deficient MPMΦ. Although we cannot exclude that altered enzymatic activity or substrate availability for ELOVL1 plays a role, a major contribution appears unlikely when considering the strongly impaired rate of degradation of VLCFA.

Taken together, these results suggest that the additional loss of *Abcd2* in *Abcd1* deficiency results in a strikingly more severe metabolic phenotype in mouse peritoneal macrophages. Although the *Abcd2* mRNA is of low abundance and was present at about only half the level of the *Abcd1* mRNA, in *Abcd1* deficiency this modest endogenous level is apparently sufficient to prevent the dramatic metabolic phenotype that we observed in *Abcd1*/*Abcd2* double-mutant macrophages. In contrast to primary mouse peritoneal macrophages, primary human monocytes/macrophages virtually lack *ABCD2* gene expression [40], [41]. Accordingly, in human X-ALD monocytes, the metabolic defects such as accumulation of VLCFA and their degradation by peroxisomal β-oxidation are comparable to those found in *Abcd1*/*Abcd2* double-deficient rather than *Abcd1*-deficient peritoneal macrophages. In MPMФ, the *Abcd2* gene is apparently not underlying the strong repression or silencing that was observed for *ABCD2* in human monocytes. Recently, we obtained evidence showing that the feasibility to induce *ABCD2* depends on the differentiation state in the human monocyte/macrophage lineage [66]. Whereas *ABCD2* was unresponsive to retinoids in primary CD14^+^ monocytes, 13-*cis*-retinoic acid produced a fourfold induction after *in vitro* differentiation into macrophages [66].

The difference between human *ABCD2* and murine *Abcd2* expression in macrophages could potentially be one contributing factor to the absence of a cerebral inflammatory phenotype in the mouse model of X-ALD. However, as also *Abcd1*/*Abcd2* double-deficient mice do not develop demyelinating brain inflammation (Forss-Petter, unpublished observation) and [31], additional species differences or other unknown triggers must play a critical role.

In conclusion, these results provide proof of principle that even a modest endogenous expression of *Abcd2* in macrophages can largely compensate for *Abcd1* deficiency and significantly reduce the amount of C26∶0 in *Abcd1*-deficient macrophages. Although these observations cannot be directly translated to human perivascular or parenchymal macrophages or activated microglia cells in brain lesions of an X-ALD patient, the results are encouraging for therapeutic strategies aiming at upregulating expression of *ABCD2*. The use of *Abcd1* and *Abcd2* single and double-deficient macrophages provides a unique model to discriminate whether ABCD2 is necessary for any observed effects of treatment in the macrophage lineage. The marked additional decrease in the capacity for β-oxidation of VLCFA upon inactivation of *Abcd2* in *Abcd1* deficiency implies a strong synergistic effect of ABCD1 and ABCD2 in this cell type.

## Supporting Information

Figure S1 ***Abcd1*** **mRNA levels in mouse peritoneal macrophages from wild-type and** ***Abcd2*** **-deficient mice.** The *Abcd1* and *Hprt* mRNA copy numbers were determined by qRT-PCR in wild-type (WT) and *Abcd2*-deficient (*Abcd2* KO) cells. The graphs indicate mean values ± SD for *Abcd1* mRNA after normalization to the level of *Hprt* mRNA in each sample (*n* = 3); *n.s*., no statistically significant difference.
(TIF)

Figure S2 **Absolute fatty acid levels in wild-type,** ***Abcd1*** **-,** ***Abcd2-*** **and** ***Abcd1/Abcd2*** **double-deficient mouse peritoneal macrophages.** The concentrations of the VLCFA species C26∶0, C24∶0 and C22∶0 and the LCFA C16∶0 were determined by GC-MS in mouse peritoneal macrophages of wild-type (WT), *Abcd1*-deficient (*Abcd1* KO), *Abcd2*-deficient (*Abcd2* KO) and *Abcd1/Abcd2* double-deficient (DOKO) mice after 5 days in culture (*n* = 3). The amounts of fatty acids: (A) C26∶0; (B) C24∶0; (C) C22∶0 and (D) C16∶0 were normalized to the protein content of each sample. The graphs indicate geometric means ± SD (asymmetrical). Statistically significant differences are indicated: * *p*<0.05, ** *p*<0.01; *n.s*., no statistically significant difference.
(TIF)

Figure S3 **Relative C26∶0 levels in wild-type,** ***Abcd1-*** **,** ***Abcd2-*** **and** ***Abcd1/Abcd2*** **double-deficient peritoneal macrophages cultured for 1-day.** The concentrations of C26∶0 and C22∶0 were determined by GC-MS in wild-type (WT), *Abcd1*-deficient (*Abcd1* KO), *Abcd2*-deficient (*Abcd2* KO) and *Abcd1/Abcd2* double-deficient (DOKO) mouse peritoneal macrophages after 1 day in culture. The relative amounts of C26∶0 are expressed as C26∶0/C22∶0 ratio. The graphs indicate geometric means ± SD (asymmetrical). Statistically significant differences are indicated: * *p*<0.05, *** *p*<0.001; (*n* = 3).
(TIF)

Figure S4 **β-Oxidation activity towards C26∶0 and C16∶0 in wild-type,** ***Abcd1-, Abcd2,*** **and** ***Abcd1/Abcd2*** **double-deficient peritoneal macrophages.** The absolute rates of β-oxidation of [^14^C]-labelled fatty acid substrates for: (A) C26∶0 and (B) C16∶0 were measured in wild-type (WT), *Abcd1*-deficient (*Abcd1* KO), *Abcd2*-deficient (*Abcd2* KO) and *Abcd1/Abcd2* double-deficient (DOKO) cells. The results are shown as the mean values ± SD of the rate (pmol/min) of released, water-soluble [^14^C]-acetyl-CoA normalized to the protein content (mg). The numbers of samples (*n*) are indicated below the graphs. Statistically significant differences are indicated: * *p*<0.05, ** *p*<0.01, *** *p*<0.001.
(TIF)

Table S1 **Absolute and normalized mRNA copy numbers of** ***Abcd1*** **,** ***Abcd2*** **and** ***Hprt*** **determined by qRT-PCR.**
(DOCX)

Table S2 **Absolute and normalized mRNA copy numbers of** ***Elovl1*** **and** ***Hprt*** **determined by qRT-PCR.**
(DOCX)
