# Supplementary figures and images for: Abcd2 Is a Strong Modifier of the Metabolic Impairments in Peritoneal Macrophages of Abcd1-Deficient Mice
Source: PLoS One. 2014 Sep 25;9(9):e108655. doi: 10.1371/journal.pone.0108655 (PMC4177892; doi:10.1371/journal.pone.0108655)

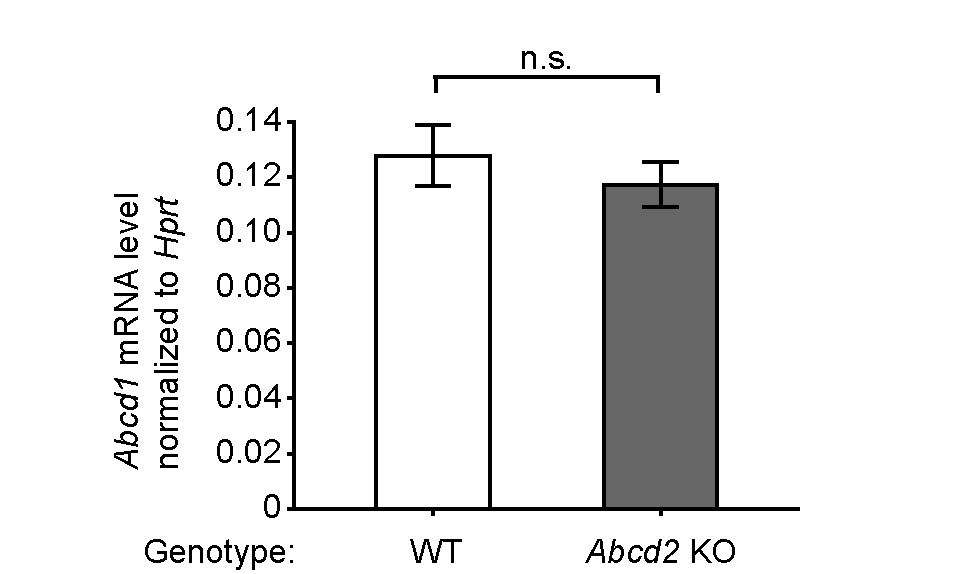

Supplement: Figure S1 — Abcd1 mRNA levels in mouse peritoneal macrophages from wild-type and Abcd2 -deficient mice. The Abcd1 and Hprt mRNA copy numbers were determined by qRT-PCR in wild-type (WT) and Abcd2-deficient (Abcd2 KO) cells. The graphs indicate mean values ± SD for Abcd1 mRNA after normalization to the level of Hprt mRNA in each sample (n = 3); n.s., no statistically significant difference. (TIF) [file pone.0108655.s001.tif]

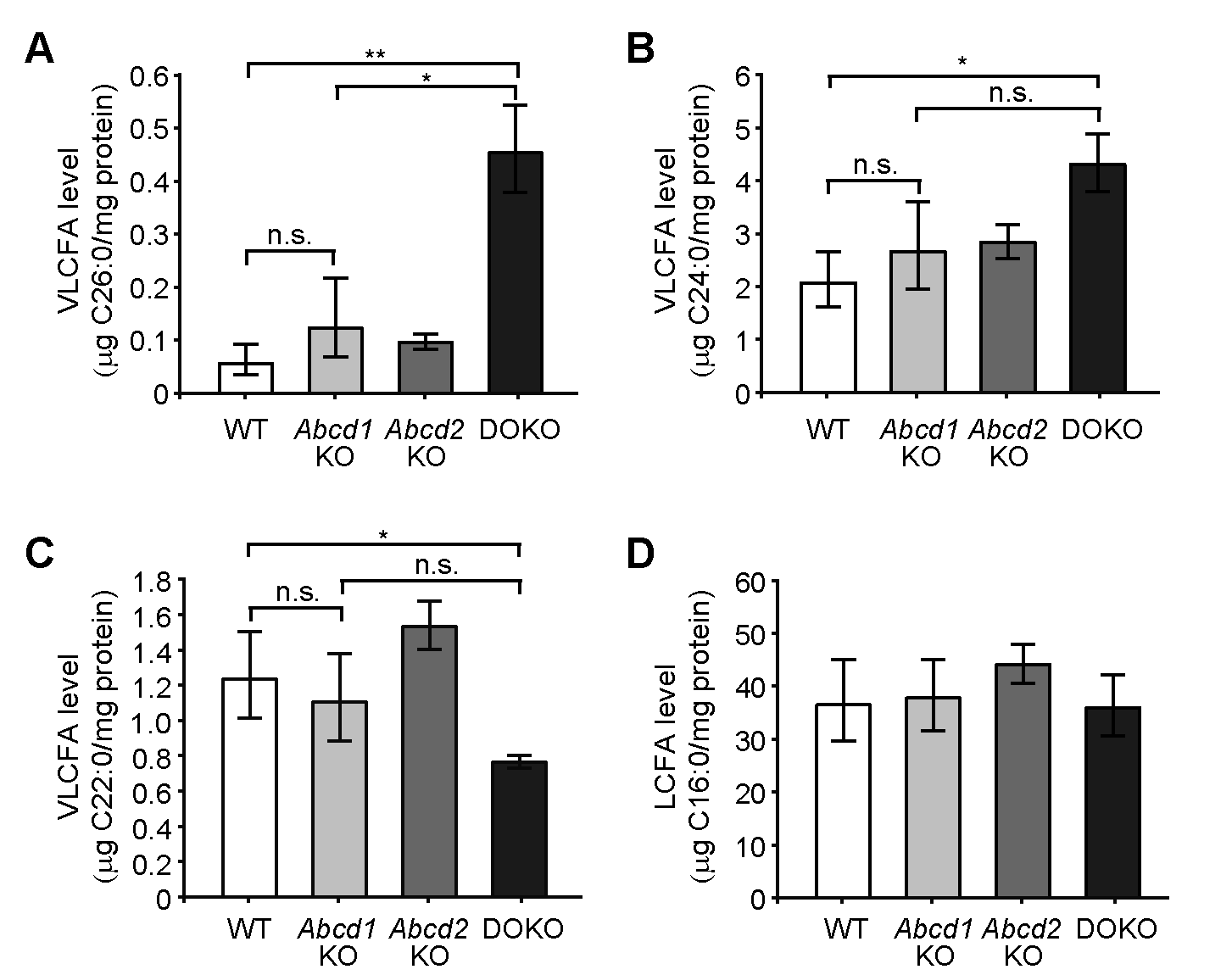

Supplement: Figure S2 — Absolute fatty acid levels in wild-type, Abcd1 -, Abcd2- and Abcd1/Abcd2 double-deficient mouse peritoneal macrophages. The concentrations of the VLCFA species C26∶0, C24∶0 and C22∶0 and the LCFA C16∶0 were determined by GC-MS in mouse peritoneal macrophages of wild-type (WT), Abcd1-deficient (Abcd1 KO), Abcd2-deficient (Abcd2 KO) and Abcd1/Abcd2 double-deficient (DOKO) mice after 5 days in culture (n = 3). The amounts of fatty acids: (A) C26∶0; (B) C24∶0; (C) C22∶0 and (D) C16∶0 were normalized to the protein content of each sample. The graphs indicate geometric means ± SD (asymmetrical). Statistically significant differences are indicated: * p<0.05, ** p<0.01; n.s., no statistically significant difference. (TIF) [file pone.0108655.s002.tif]

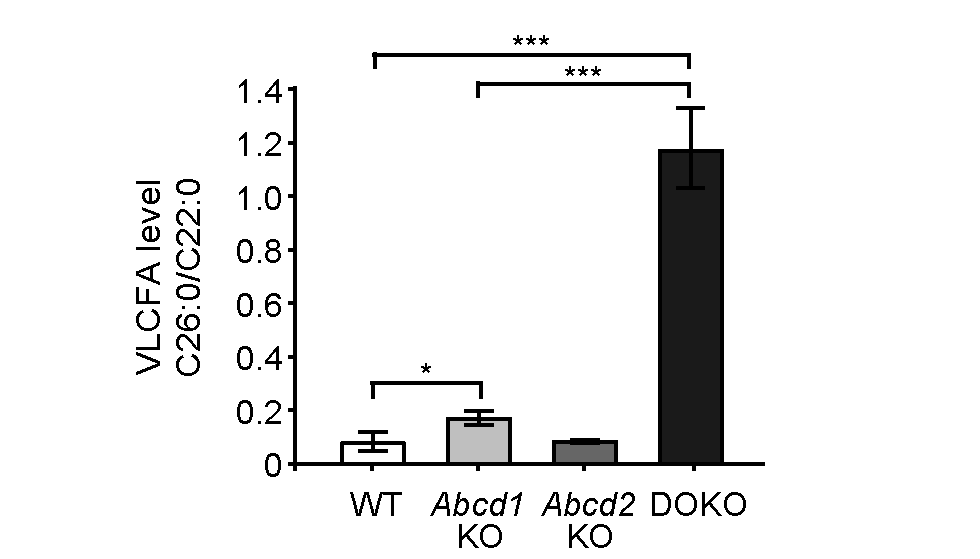

Supplement: Figure S3 — Relative C26∶0 levels in wild-type, Abcd1- , Abcd2- and Abcd1/Abcd2 double-deficient peritoneal macrophages cultured for 1-day. The concentrations of C26∶0 and C22∶0 were determined by GC-MS in wild-type (WT), Abcd1-deficient (Abcd1 KO), Abcd2-deficient (Abcd2 KO) and Abcd1/Abcd2 double-deficient (DOKO) mouse peritoneal macrophages after 1 day in culture. The relative amounts of C26∶0 are expressed as C26∶0/C22∶0 ratio. The graphs indicate geometric means ± SD (asymmetrical). Statistically significant differences are indicated: * p<0.05, *** p<0.001; (n = 3). (TIF) [file pone.0108655.s003.tif]

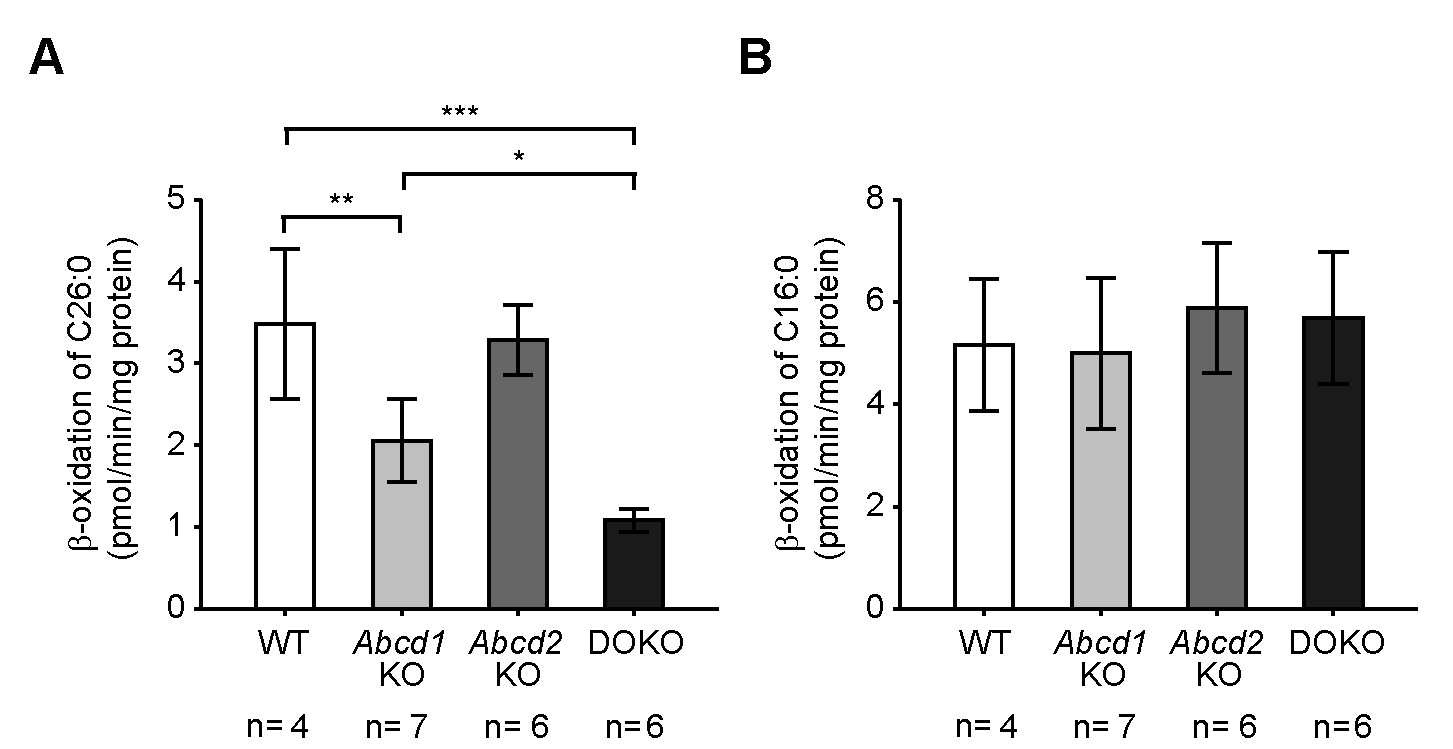

Supplement: Figure S4 — β-Oxidation activity towards C26∶0 and C16∶0 in wild-type, Abcd1-, Abcd2, and Abcd1/Abcd2 double-deficient peritoneal macrophages. The absolute rates of β-oxidation of [14C]-labelled fatty acid substrates for: (A) C26∶0 and (B) C16∶0 were measured in wild-type (WT), Abcd1-deficient (Abcd1 KO), Abcd2-deficient (Abcd2 KO) and Abcd1/Abcd2 double-deficient (DOKO) cells. The results are shown as the mean values ± SD of the rate (pmol/min) of released, water-soluble [14C]-acetyl-CoA normalized to the protein content (mg). The numbers of samples (n) are indicated below the graphs. Statistically significant differences are indicated: * p<0.05, ** p<0.01, *** p<0.001. (TIF) [file pone.0108655.s004.tif]
