# Supplementary material for: Abcd2 Is a Strong Modifier of the Metabolic Impairments in Peritoneal Macrophages of Abcd1-Deficient Mice
Source: PLoS One. 2014 Sep 25;9(9):e108655. doi: 10.1371/journal.pone.0108655 (PMC4177892; doi:10.1371/journal.pone.0108655)
Supplement: Table S1 — Absolute and normalized mRNA copy numbers of Abcd1 , Abcd2 and Hprt determined by qRT-PCR. (DOCX) [file pone.0108655.s005.docx]

**Table S1: Absolute and normalized mRNA copy numbers of *Abcd1*, *Abcd2* and *Hprt* determined by qRT-PCR.**

|  | | **mRNA S.Q. ^1^** | | | **S.Q. normalized to *Hprt*** | | | |
| --- | --- | --- | --- | --- | --- | --- | --- | --- |
| **Genotype** | **Sample No.** | ***Abcd1*** | ***Abcd2*** | ***Hprt*** | ***Abcd1*/*Hprt*** | ***Abcd1*/*Hprt* (mean)** | ***Abcd2*/*Hprt*** | ***Abcd2*/*Hprt* (mean)** |
| **Wild-type** | 1 | 2.19 x 10^3^ | 1.46 x 10^3^ | 1.51 x 10^4^ | 0.145 | 0.128 | 0.097 | 0.075 |
|  | 2 | 1.35 x 10^3^ | 8.09 x 10^2^ | 1.08 x 10^4^ | 0.125 |  | 0.075 |  |
|  | 3 | 2.36 x 10^3^ | 1.53 x 10^3^ | 1.91 x 10^4^ | 0.124 |  | 0.080 |  |
|  | 4 | 2.21 x 10^3^ | 1.20 x 10^3^ | 1.70 x 10^4^ | 0.130 |  | 0.071 |  |
|  | 5 | 2.75 x 10^3^ | 1.21 x 10^3^ | 2.38 x 10^4^ | 0.116 |  | 0.051 |  |
| ***Abcd1* KO** | 6 | n.d.^2^ | 1.84 x 10^3^ | 1.78 x 10^4^ | n.d. | n.d. | 0.103 | 0.095 |
|  | 7 |  | 1.57 x 10^3^ | 1.53 x 10^4^ |  |  | 0.103 |  |
|  | 8 |  | 1.29 x 10^3^ | 1.61 x 10^4^ |  |  | 0.080 |  |
| ***Abcd2* KO** | 9 | 3.60 x 10^3^ | n.d. | 2.90 x 10^4^ | 0.124 | 0.117 | n.d. | n.d. |
|  | 10 | 2.61 x 10^3^ |  | 2.41 x 10^4^ | 0.108 |  |  |  |
|  | 11 | 2.36 x 10^3^ |  | 1.98 x 10^4^ | 0.119 |  |  |  |

1) S.Q., Starting Quantity, initial cDNA copy number derived from 4 ng total RNA

2) n.d., not determined
