# Supplementary material for: Abcd2 Is a Strong Modifier of the Metabolic Impairments in Peritoneal Macrophages of Abcd1-Deficient Mice
Source: PLoS One. 2014 Sep 25;9(9):e108655. doi: 10.1371/journal.pone.0108655 (PMC4177892; doi:10.1371/journal.pone.0108655)
Supplement: Table S2 — Absolute and normalized mRNA copy numbers of Elovl1 and Hprt determined by qRT-PCR. (DOCX) [file pone.0108655.s006.docx]

**Table S2: Absolute and normalized mRNA copy numbers of *Elovl1* and *Hprt* determined by qRT-PCR.**

|  | | **mRNA S.Q. ^1^** | | **S.Q. normalized to *Hprt*** | |
| --- | --- | --- | --- | --- | --- |
| **Genotype** | **Sample No.** | ***Elovl1*** | ***Hprt*** | ***Elovl1/Hprt*** | ***Elovl1/Hprt* (mean)** |
| **Wild-type** | 3 | 4.39 x 10^3^ | 2.05 x 10^4^ | 0.214 | 0.280 |
|  | 4 | 4.64 x 10^3^ | 1.53 x 10^4^ | 0.304 |  |
|  | 5 | 7.09 x 10^3^ | 2.19 x 10^4^ | 0.323 |  |
| ***Abcd1* KO** | 6 | 5.87 x 10^3^ | 2.56 x 10^4^ | 0.229 | 0.272 |
|  | 7 | 7.20 x 10^3^ | 2.47 x 10^4^ | 0.291 |  |
|  | 8 | 7.71 x 10^3^ | 2.60 x 10^4^ | 0.297 |  |
| ***Abcd2* KO** | 9 | 6.97 x 10^3^ | 3.00 x 10^4^ | 0.232 | 0.266 |
|  | 10 | 7.47 x 10^3^ | 2.61 x 10^4^ | 0.286 |  |
|  | 11 | 6.29 x 10^3^ | 2.26 x 10^4^ | 0.279 |  |
| **DOKO** | 12 | 5.78 x 10^3^ | 2.24 x 10^4^ | 0.258 | 0.260 |
|  | 13 | 6.05 x 10^3^ | 2.62 x 10^4^ | 0.231 |  |
|  | 14 | 6.80 x 10^3^ | 2.35 x 10^4^ | 0.290 |  |

1) Mean Starting Quantity, initial cDNA copy number derived from 4 ng total RNA
